# Exploring the barriers to pulmonary rehabilitation for patients with chronic obstructive pulmonary disease: a qualitative study

**DOI:** 10.1186/s12913-021-06814-5

**Published:** 2021-08-17

**Authors:** Ramin Sami, Kobra Salehi, Marzieh Hashemi, Vajihe Atashi

**Affiliations:** 1 Department of Internal Medicine, School of Medicine, Isfahan University of Medical Science, Isfahan, Iran; 2 Department of Midwifery and Reproductive Health, Nursing and Midwifery Care Research Center, School of Nursing and Midwifery, Isfahan University of Medical Science, Isfahan, Iran; 3 Adult Health Nursing Department, Nursing and Midwifery Care Research Center, School of Nursing and Midwifery, Isfahan University of Medical Science, Isfahan, Iran

**Keywords:** Pulmonary rehabilitation, Barriers, Chronic obstructive pulmonary disease, Qualitative study

## Abstract

**Background:**

The complexity of chronic obstructive pulmonary disease (COPD) and its different physical, mental, familial, occupational, and social complications highlight the necessity of pulmonary rehabilitation (PR) for afflicted patients. However, PR for patients with COPD usually faces some barriers. The aim of this study was to explore the barriers to PR for patients with COPD.

**Methods:**

This qualitative descriptive study was conducted in January 2019 to October 2020. Participants were 19 patients with COPD, 11 family caregivers of patients with COPD, and 12 healthcare providers, who all were recruited purposively from two teaching hospitals in Isfahan, Iran. Data were collected through semi-structured interviews and were analyzed through conventional content analysis.

**Results:**

The barriers to PR for patients with COPD fell into three main categories, namely barriers related to patients and their families, inefficiency of PR services, and inappropriate organizational context for PR. Each category had four subcategories, namely patients’ and families’ lack of knowledge, complexity and chronicity of COPD, heavy financial burden of COPD, patients’ frustration and discontinuation of PR, lack of patient-centeredness, lack of coordination in PR team, inadequate professional competence of PR staff_,_ lack of a holistic approach to PR, limited access to PR services, inadequate insurance for PR services, ineffective PR planning, and discontinuity of care.

**Conclusion:**

PR for patients with COPD is a complex process which faces different personal, familial, social, financial, organizational, and governmental barriers. Strategies for managing these barriers are needed in order to improve the effectiveness and the quality of PR services for patients with COPD.

**Supplementary Information:**

The online version contains supplementary material available at 10.1186/s12913-021-06814-5.

## Background

Chronic obstructive pulmonary disease (COPD) is one of the most prevalent chronic diseases in the world [1]. By definition, COPD refers to a series of conditions, including chronic bronchitis and emphysema, which are associated with the chronic obstruction of air flow in the airways. Airway obstruction is a diffused stenosis in the airways which increases the resistance against airflow [2]. Airflow limitation in the airways in COPD is usually irreversible and characterized by symptoms such as dyspnea, cough, and sputum [3, 4]. Currently, there are around 80 million people with COPD in the world [5]. There are no reliable statistics on the prevalence of COPD in Iran though a study in five provinces reported a prevalence of 4.9% [2].

COPD is associated with many different complications ranging from dyspnea with no significant effects on activity level to complete inability to perform daily activities due to dyspnea [6]. Statistics show that more than 70% of patients with COPD have problems in doing daily activities and patients with severe COPD even experience dyspnea and early fatigue during simple walking at home [7]. Accordingly, COPD may result in inability to effectively perform social roles, dependence on others, and mental health problems such as fear, anxiety, depression, and isolation [8]. COPD is currently the fourth leading cause of death and is estimated to turn into the third leading cause of death by 2030 [4].

Pulmonary rehabilitation (PR) is one of the main components of COPD management [9]. According to the American Thoracic Society and the European Thoracic Society, PR is a comprehensive intervention based on the careful assessment of patients accompanied by patient-centered care including exercise training and behavior modification in order to improve patients’ physical and mental status [10]. The main goals of PR are to maintain patient autonomy, prevent complications, promote physical functioning, restore self-confidence, enhance life satisfaction, and help patients return to society [11, 12]. PR also alleviates respiratory symptoms [13], improves quality of life [13, 14], and boosts activity level [14]. A study also showed that PR reduced dyspnea by 93%, reduced the severity of fatigue-related symptoms by 73%, and alleviated pain by 74% [15].

Despite the known benefits of PR for patients with COPD, studies show that there are many barriers to PR [16]. For instance, two studies reported inadequate education respecting COPD and prevention of its complications, limited mental support, lack of knowledge, and poor interdisciplinary coordination respecting PR as the main barriers to PR [17, 18]. Another study found that the main barriers to PR were poor coping with COPD, limited access to PR services, high levels of anxiety, and dependence on others [16].

A major factor affecting PR is culture [19, 20]. To be successful in pulmonary rehabilitation programs, not only act evidence based, but also pay attention to the context and structure that program is run [21]. Although pulmonary rehabilitation is a global standard intervention, in practice the structural details of the program will vary somewhat based on care systems and socio-cultural differences. If pulmonary rehabilitation programs do not remove the barriers, these services may not be able to meet the needs of patients [22]. Therefore, studies, particularly with qualitative designs, are needed in different cultures in order to provide deeper understanding about barriers to PR [23]. Nonetheless, our literature search revealed that most studies into the barriers to PR in Iran were conducted using quantitative designs which assessed limited PR-related variables and did not provide a deep understanding about the barriers to PR [24, 25]. Therefore, the present study was conducted to narrow this gap. The aim of the study was to explore the barriers to PR for patients with COPD.

## Methods

This qualitative descriptive study was conducted in January 2019 to October 2020.

### Participants and setting

Study setting was the lung care wards of Al-Zahra and Khorshid hospitals as well as the Comprehensive Respiratory Clinic of Khorshid hospital, Isfahan, Iran. The clinic is a PR center for patients with chronic pulmonary diseases and was established in 2018. Healthcare providers in this clinic are physicians, nurses, nutritionists, physical therapists, social workers, psychologists, and psychiatrists. Patients are referred to this clinic by physicians where they receive PR services and education about nutrition, physical exercise, and self-care.

Participants were 19 patients with COPD, 11 family caregivers of patients with COPD, and 12 healthcare providers—42 in total. Sampling was purposively performed with maximum variation regarding age, gender, education level, and occupation. Inclusion criteria for patients were a definite diagnosis of stage 2–4 COPD at least 1 year before the study, ability to establish verbal communication, no hearing impairment, no cognitive or mental disorder such as severe depression, and agreement for participation in the study. COPD diagnosis was established based on spirometry findings and the criteria of the Global Initiative for Chronic Obstructive Lung Disease (GOLD) [26] (Table 1). Inclusion criteria for other participants were the experience of care delivery to patients with COPD for at least 2 years and agreement for participation.

**Table 1:** Participants’ characteristics

No	Age	Gender	Occupation	Educational level	COPD severity^a^	Caregiving experience
1	48	Female	Housewife	Primary	Stage 2	–
2	50	Female	Housewife	Primary	Stage 4	–
3	57	Male	Employee	Secondary	Stage 3	–
4	59	Female	Housewife	Illiterate	Stage 3	–
5	56	Male	Self-employed	Primary	Stage 3	–
6	60	Male	Self-employed	Secondary	Stage 3	–
7	55	Male	Employee	Diploma	Stage 2	–
8	63	Male	Retired	Primary	Stage 4	–
9	68	Male	Retired	Associate diploma	Stage 4	–
10	70	Male	Retired	Secondary	Stage 4	–
11	75	Male	Retired	Illiterate	Stage 4	–
12	66	Male	Unemployed	Secondary	Stage 3	–
13	70	Male	Retired	illiterate	Stage 2	–
14	74	Male	Retired	Primary	Stage 3	–
15	69	Female	Retired	Diploma	Stage 3	–
16	58	Male	Self-employed	secondary	Stage 3	–
17	82	Male	Unemployed	Illiterate	Stage 4	–
18	78	Male	Retired	Diploma	Stage 3	–
19	73	Male	Self-employed	Secondary	Stage 3	–
20	56	Female	Housewife	Diploma	–	5 years
21	32	Female	Employee	Associate diploma	–	6 years
22	38	Female	Housewife	Diploma	–	3 years
23	39	Female	Employee	Bachelor’s	–	8 years
24	45	Female	Housewife	Secondary	–	6 years
25	43	Female	Housewife	Diploma	–	3 years
26	52	Female	Self-employed	Diploma	–	6 years
27	58	Female	Housewife	Primary	–	4 years
28	60	Male	Retired	Secondary	–	9 years
29	42	Male	Self-employed	Bachelor’s	–	7 years
30	70	Male	Retired	Secondary	–	10 years
31	45	Male	pulmonologist	Subspecialist	–	8 years
32	37	Female	pulmonologist	Subspecialist	–	3 years
33	50	Male	Psychiatrist	Specialist	–	20 years
34	39	Female	Nutritionist	Bachelor’s	–	2 years
35	45	Male	Physical therapist	Bachelor’s	–	6 years
36	39	Female	Physical therapist	Master’s	–	4 years
37	28	Female	Nurse	Bachelor’s	–	5 years
38	36	Female	Nurse	Master’s	–	8 years
39	26	Female	Nurse	Bachelor’s	–	6 years
40	37	Female	Psychologist	Master’s	–	2 years
41	45	Female	PR specialist	Specialist	–	8 years
42	32	Female	Social worker	Bachelor’s	–	3 years

^a^: COPD severity was determined based on the GOLD criteria as follows:

Stage 2: FEV1/FVC < 70%;FEV1: 50–79%

Stage 3: FEV1/FVC < 70%; FEV1: 30–49%

Stage 4: FEV1/FVC < 70%; FEV1 < 30%

### Data collection

Data were collected via semi-structured personal interviews. The time and the place of the interviews were determined according to participants’ preferences and the length of the interviews was 30–90 min, depending on participants’ conditions and willingness to share their experiences. Interviews were guided using an interview guide, the validity of which was approved by three experts in qualitative research. Moreover, the interview guide was piloted in two primary interviews which resulted in no significant revision to the guide. During interviews, participants were asked and encouraged to share their experiences regarding the barriers to PR. Examples of interview questions for patients with COPD and their family caregivers were, “What measures have you taken so far to promote your recovery?”, “What measures do you take during caregiving to your patient?”, and “What problems did you face during PR?”. Examples of interview questions for participating healthcare providers were, “What care measures do you take for patients with COPD?”, “How is PR for patients with COPD performed?”, and “What are the barriers to PR for patients with COPD?”. Follow-up questions were also used based on participants’ responses to main interview questions. Examples of these questions were, “What do you mean by this?”, “Can you provide further explanation about this?”, “Why did you that?”, and “How did you that?”. Moreover, participants were asked to provide examples of their experiences or explain about the reasons of their actions or responses. Data collection was continued until the point of data saturation, i.e., when the new data became repetitive. Interviews were audio-recorded, though three participants did not consent for audio-recording and hence, their interviews were manually documented.

### Data analysis

Data analysis was performed concurrently with data collection using Graneheim and Lundman approach to conventional content analysis. Initially, interviews were transcribed word by word and reviewed for several times in order to acquire a general understanding about the data. Then, each interview transcript was carefully read word by word and words and expressions which were related to the study aim were identified as meaning units and coded. Codes were grouped into subcategories according to their similarities. Subcategories were also grouped into larger categories according to their similarities and interrelationships [27].

### Trustworthiness

The trustworthiness of the findings was ensured using the credibility, dependability, conformability, and transferability criteria [28]. Conformability was ensured using external checking, through which excerpts from the data together with their codes, subcategories, and categories were provided to two qualitative researchers who assessed and approved the accuracy of data analysis. Moreover, all steps of the study were documented in detail in order to provide others with the opportunity to trace them. Sampling with maximum variation respecting participants’ age, gender, educational level, and occupation helped establish transferability. Credibility was ensured through member checking and dependability was ensured through concurrent data collection and analysis.

### Findings

In total, 42 patients with COPD, family caregivers of patients with COPD, and healthcare providers participated in the study. Table 1 shows their characteristics.

Participants’ experiences of the barriers to PR for patients with COPD were categorized into three main categories, namely barriers related to patients and their families, inefficiency of PR services, and inappropriate organizational context for PR (Table 2).

**Table 2:** The main categories and subcategories of the study

Subcategories	Categories
Patients’ and families’ lack of knowledge	Barriers related to patients and their families
Complexity and chronicity of COPD	
Heavy financial burden of COPD	
Patients’ frustration and discontinuation of PR	
Lack of patient-centeredness	Inefficiency of PR services
Lack of coordination in PR team	
Inadequate professional competence of PR staff	
Lack of a holistic approach to PR	
Limited access to PR services	Inappropriate organizational context for PR
Inadequate insurance for PR services	
Ineffective PR planning	
Discontinuity of care	

#### Barriers related to patients and their families

Compared with PR for patients with other chronic diseases, PR for patients with COPD is more difficult and complex due to the complexity and chronicity of COPD, patients’ and families’ lack of knowledge, heavy financial burden of COPD management, and patients’ frustration and discontinuation of PR.

##### Patients’ and families’ lack of knowledge

Most participating patients and family caregivers highlighted that COPD had completely been unknown for them and they had no information about it at the time of diagnosis. Therefore, they noted that they had to obtain more information about the disease before taking any other measure after receiving diagnosis.

*I needed information about how to care for my husband to help him not experience so severe dyspnea. I didn’t know anything about his appropriate nutrition and his independent performance of daily activities. Nonetheless, I couldn’t find any information about this (P. 13).*

##### Complexity and chronicity of COPD

COPD is a chronic disease which necessitates long-term treatment and care. Moreover, some afflicted patients are dependent on oxygen therapy and COPD has courses of symptom remission and exacerbation which may result in hospitalization. All these factors may interfere with PR.

*I can’t walk at all and am dependent on oxygen. I can’t go out of home. I easily get tired even when I walk several steps at home. How can I refer to the clinic with such conditions? (P. 3).*

##### Heavy financial burden of COPD

The chronicity of COPD, the necessity for continuous treatment and care, and the high costs of medications and PR services caused patients and their families heavy financial strain and burden. On the other hand, the stress and concern related to the inability to buy medications and receive PR aggravated their conditions.

*Many patients and their families do not continue PR. They have right to discontinue PR because they should pay for many different services such as physical therapy, medications, transportation, and specific foods in this bad economic condition of the country (P. 41).*

##### Patients’ frustration and discontinuation of PR

While family caregivers and healthcare providers attempted to provide quality PR to patients, actualize their potentials, and promote their autonomy, patients’ frustration caused difficulties in PR and made attempts fruitless. The causes of patients’ frustration were the psychological burden imposed by family members, inability to effectively cope with COPD, and inability to return to an independent life.

*I know I never achieve recovery and will finally die of this disease. Then, why should I receive rehabilitation and physical therapy? These interventions and exercises never recover my lung. The damage was done. (P.17)*

#### Inefficiency of PR services

The second main category of the study was the inefficiency of PR services which refers to limited interdisciplinary coordination among the members of PR team, limited attention to patients and their real needs, and limited professional competence of PR staff. The four subcategories of this category were lack of patient-centeredness, lack of coordination in PR team, inadequate professional competence of PR staff, and lack of holistic approach to PR.

##### Lack of patient-centeredness

The core of PR is patient-centeredness. However, most participating patients and family caregivers noted that while making PR-related decisions, PR staff did not consider patients’ preferences and needs and even did not perform a careful patient assessment. They expected PR staff to involve them in PR-related decision making and pay attention to their experiences and words.

*Unfortunately, we don’t have a holistic approach to patients in PR due to poor interpersonal interactions. For example, a patient may complain of inadequate sleep but nobody asks about his/her daily diet and activities. Patient assessment is not performed correctly. They simply prescribe sleep medications while the patients may have developed sleeplessness due to the intake of theophylline or excessive tea (P. 37).*

##### Lack of coordination in PR team

Most participants referred to the lack of coordination among PR staff as a major barrier to PR. Strong interactions and close collaboration among PR staff are among the key factors contributing to the success of PR and their lack can result in the inefficiency of PR, exacerbation of patients’ problems, and imposition of heavy costs on patients and healthcare systems.

*We have no relationships with each other. Now we are working as a team. Therefore, we need to have joint patient rounds and assessments. Of course, I haven’t so far heard that the members of a healthcare team hold a meeting to talk about a patient. (P.41)*

#### Inadequate professional competence of PR staff

Given the critical importance of PR to patient outcomes, PR staff should have great professional competence. According to the participants, professional competence for PR staff includes professional accountability, competence in quality patient education, great professional knowledge, motivation for patient care, and sense of belonging to PR team. However, participants noted that PR staff did not have great professional competence for PR.

*As a nurse, I haven’t received any education about PR so far and hence, I can’t accurately assess a patient and determine his/her needs. I just provide a series of general educations to all patients while patients’ needs differ from each other. Here, we just perform a series of routine tasks such as education about pursed-lip breathing. This can’t be called PR. (P.39)*

##### Lack of a holistic approach to PR

In the process of PR, each patient should be considered as a bio-psycho-socio-spiritual being and all these aspects should be addressed because they affect each other. However, participants’ experiences showed that limited attention, if any, is paid to the different aspects of PR.

*I cough a lot and have dyspnea and hence, I have developed sexual dysfunction. I’m ill but my wife, who is fifty year-old, is not ill. I feel she thinks I am impotent. Nobody assesses this problem. Whom should I tell about this? I have a bad feeling about it (P. 7).*

#### Inappropriate organizational context for PR

According to the participants, the inappropriate organizational context of the healthcare system makes difficulties in PR. This category had four subcategories, namely limited access to PR services, inadequate insurance for PR services, ineffective PR planning, and discontinuity of care.

##### Limited access to PR services

Some patients with COPD have limited access to PR services due to the unavailability of PR services in the evening and the night shifts, their long distance to PR centers, inadequate number of PR centers, lack of PR centers in some cities and even provinces, limited PR equipment in some PR centers, and limited governmental and social support for afflicted patients.

*This is a major challenge that we have no center or few centers for PR in this large city with this large number of patients who need PR. We even don’t have adequate equipment in the existing centers. These patients need specialized equipment for PR but unfortunately nobody cares (P. 36).*

##### Inadequate insurance for PR services

Patients and family caregivers highlighted that insurance companies just covered some costs of the medications manufactured in Iran and did not pay for the imported medications. Meanwhile, most of them reported that medications manufactured in Iran did not effectively alleviate their symptoms. Moreover, they noted that despite their serious need for PR services, insurance companies never covered services such as physical therapy and professional counseling.

*The costs of the imported medications have increased due to the exchange rate fluctuations and hence their purchase has become very difficult for many low-income patients. Living conditions have become really difficult and troublesome. I wish insurance companies provided more support for buying medications (P. 22).*

##### Ineffective PR planning

Effective organization and planning are important factors for quality PR. Nonetheless, participants’ experiences showed defects in PR organization and planning due to problems such as lack of clear job description for PR staff, lack of clear PR protocols, and inefficiency of the PR record system.

*We don’t have any guideline and don’t know what we should do for patients with COPD who come to receive PR. Sometimes, we see duplication of efforts due to the lack of a clear job description and sometimes we don’t know at all what we should do and how to plan for next patient visits (P. 32).*

##### Discontinuity of care

Lack of follow-up assessment and services outside healthcare settings and lack of supervision on PR services break care continuity. Participants’ experiences showed that only those patients who could refer to PR centers could receive PR services. In other words, patients who could not go out of home due to problems such as severe physical problems or lack of family support could not receive PR services. Most participating patients, family caregivers, and healthcare providers were dissatisfied with the lack of follow-up services in non-clinical and home settings.

*Most of our patients complain about the lack of follow-up services and the sense of abandonment. The limitation of our services to the clinic is not useful. We should extend our services to home settings. Many problems of patients should be assessed in home settings (P. 42).*

## Discussion

This study explored the barriers to PR for patients with COPD. Findings showed that the major barriers to PR for patients with COPD were barriers related to patients and their families, inefficiency of PR services, and inappropriate organizational context for PR. These findings imply that the barriers to PR for patients with COPD are personal, familial, social, financial, organizational, and governmental.

One of the main barriers to PR for patients with COPD was the barriers related to patients and their families. Most participants reported the characteristics of patients and COPD as barriers to PR. In line with this finding, a former study revealed COPD severity and patients’ age as barriers to PR [17]. Financial burden related to medications and PR services was another barrier to PR in the present study. Generally, the costs of treatment, medications, care, and PR impose heavy financial burden on patients with COPD. Financial inability to receive PR services may result in greater disability, loss of employment, or mandatory retirement, particularly among patients with severer COPD and greater disability. Therefore, adequate financial support for these patients is needed to alleviate their conditions, prevent their disability and loss of employment, and thereby, reduce the personal and social costs of COPD [29].

We also found psychological burden imposed by family members, inability to effectively cope with COPD, and dependence on others in doing daily activities as barriers to PR for patients with COPD. Similarly, a former study showed that the effectiveness of treatment, care, and PR for patients with COPD largely depends on their effective coping with COPD [30]. Another study highlighted that effective PR requires emotional support and hope giving by family members [31]. Because of special close family relationship in Iranian culture, providing sufficient information about pulmonary rehabilitation, essential training and education to the patient’s family can be useful and effective [32].

Inefficiency of PR services was the second main barrier to PR in the present study. Findings revealed poor interactions in PR team, lack of patient-centeredness, and lack of a holistic approach to PR as factors contributing to the inefficiency of PR services. These factors resulted in isolated goal setting and PR delivery which in turn increased PR-related costs and caused problems and complications for patients. In line with these findings, an earlier study showed that poor communication and coordination, lack of financial resources, lack of knowledge about interdisciplinary collaboration, lack of knowledge about PR-related measures taken by other healthcare providers, and limited experience in PR delivery reduced the effectiveness of PR for patients with asthma [33]. Similarly, another study reported that interpersonal relationships in interdisciplinary teams are weak and may be complicated by some presumptions such as the superiority of some professions over others which eventually result in the reduced effectiveness of PR [34]. Teamwork in healthcare settings in Iran is still poor and hence, healthcare authorities need to take serious measures for teamwork promotion, particularly in PR teams.

Patients’ and family members’ non-involvement in the process of decision making for PR was another barrier to PR in the inefficiency of PR services main category. In recent years, healthcare authorities in Iran strongly emphasized on the involvement of patients and their families in clinical decision-making as well as the necessity of patient-centered care [35]. A study reported that strategies such as quality patient education and providing patients with the opportunity to express their ideas and recommendations are effective for encouraging their involvement in the process of clinical decision making [18]. Pulmonary rehabilitation service personnel should devote sufficient time to patients and provide them with necessary support to enhance active patient and family involvement. Compassion-based interventions strengthen trust and security in patients and families and increase their active participation [36].

Our findings also showed inadequate professional competence of PR staff as another factor contributing to the inefficiency of PR services. All members of PR team should have technical and communicative competence and receive the necessary education for the delivery of effective PR services [37]. Moreover, we found lack of holistic approach to PR as a barrier to PR for patients with COPD. In other words, while patients with COPD have different bio-psycho-socio-spiritual needs, participants reported that PR staff mainly focused on the fulfillment of physical needs. Contrarily, PR services in some developed countries are more coherent and organized and address different needs of patients [38, 39]. In most chronic diseases, including COPD, sexual function is affected and the likelihood of sexual dysfunction increases following simultaneous and multiple illnesses [40]. Patients stated that talking about sex with their caregivers was unpleasant and that caregivers were reluctant to talk to the patient about it. Talking about sex is still a cultural taboo in our country. The shame of revelation stemming from the culture and religious beliefs of the Iranian people about sexual issues causes not only the members of the rehabilitation team not to be educated in this field, but also the sick and their families to refrain from asking questions in this field. This finding shows the need for active care and attention of caregivers regarding the performance and sexual satisfaction of patients and a comprehensive study of patients in various dimensions. The health team needs to take the lead so that patients can talk about sex. Sexual health should be considered as an important part of patient evaluation [41].

The third main category of the barriers to PR for patients with COPD was inappropriate organizational context for PR. One of the subcategories of this category was patients’ limited access to PR services. Home visit for improving home environment and providing correct home-based services is a key component of PR for patients with COPD [42]. However, home visit is not performed in many developing countries, like Iran, resulting in patients’ limited access to some PR services and reduced effectiveness of PR [43].

Inadequate insurance for PR services was another barrier to PR in the inappropriate organizational context for PR main category. COPD management bears the heavy costs of medications, PR, and counseling services which are mostly covered in developed countries by insurance companies [44, 45]. However, insurance companies in Iran do not cover the majority of the costs of COPD management including the costs of PR. Therefore, providing COPD-afflicted patients with stronger financial support [43] and improving the quality and accessibility of PR services are needed to improve the effectiveness of PR services [46].

In low- and middle-income countries, where pulmonary rehabilitation centers are limited and difficult accessed, some of these interventions can be done at home. Home weights such as water bottles can also be used for resistance training at home [47]. Traditional rituals such as local and indigenous dances in physical exercise are another Solution for pulmonary rehabilitation programs that will increase patient acceptance, access, and participation in low income countries. These include the use of traditional dances or yoga and tai chi in India, Vietnam and Kyrgyzstan. It is possible to implement or monitor some of these rehabilitation programs from existing technologies such as the use of smartphone programs or online sites, and the necessary awareness in this regard can be increased through information campaigns. It is also possible to schedule home visits for patients who do not have access to pulmonary rehabilitation services, and for the patient to receive the necessary training at home and be followed up by telephone [48].

### Strengths of the study

This is among the handful of studies in Iran which explored the barriers to PR for patients with COPD. Participation of patients, family caregivers, and different healthcare providers, including physicians, nurses, psychologists, and physical therapies, was a main strength of the study.

### Limitations

Two participants did not consent for audio-recording their interviews and hence, we had to rapidly make notes of their interviews which resulted in the missing of some parts of their data. Also the study heavily responds to the practice in Iran that is radically different to other parts of the world and that limitation in generalizability.

## Conclusion

This study suggests that PR for patients with COPD is a complex process which faces different personal, familial, social, financial, organizational, and governmental barriers. Ineffective management of these barriers can result in patients’ frustration, abandonment of PR, and aggravation of their conditions. Healthcare authorities and patients’ families need to provide COPD-afflicted patients with greater support in order to improve their access to quality PR services and thereby reduce their physical and mental problems and improve their conditions.

## Supplementary Information

**Additional file 1.** Interview guide.

## Data Availability

The datasets analyzed during the current study available from the corresponding author on reasonable request.

## Abbreviations

COPD: Chronic Obstructive Pulmonary Disease
PR: Pulmonary Rehabilitation
GOLD: Global Initiative of Chronic Lung Disease
