# Supplementary material for: Exploring the barriers to pulmonary rehabilitation for patients with chronic obstructive pulmonary disease: a qualitative study
Source: BMC Health Serv Res. 2021 Aug 17;21:828. doi: 10.1186/s12913-021-06814-5 (PMC8369747; doi:10.1186/s12913-021-06814-5)
Supplement: Supplementary file 1 — Additional file 1. Interview guide. [file 12913_2021_6814_MOESM1_ESM.docx]

**Interview guide**

**Interview guide for patients/family caregivers**

How much has COPD affected your daily life?

What measures have you taken so far to promote your recovery? / What measures have you taken so far to promote your patient’s recovery?

How much information do you have about PR? Do you have any experience of PR delivery?

What are your PR-related goals and expectations?

Do your caregivers/family members support you to participate in PR programs?

What problems and barriers have you experienced with respect to PR?

**Interview guide for healthcare providers**

“What care measures do you take for patients with COPD?”

“How is PR for patients with COPD performed?”

“What are the main aspects of PR for patients with COPD?”

“What are the barriers to PR for patients with COPD?”.

How do you guide your patients to set their PR-related goals?

Do you involve patients’ families in PR?

What is the role of patients’ families in PR?

What is the role of healthcare team in PR?

What are the characteristics of a good PR team?

In your opinion, why some patients do not continue their PR program?

What problems and barriers have you experienced with respect to PR?
